# Modulation of Gut Microbiota, and Morphometry, Blood Profiles and performance of Broiler Chickens Supplemented with *Piper aduncum*, *Morinda citrifolia*, and *Artocarpus altilis* leaves Ethanolic Extracts

**DOI:** 10.3389/fvets.2024.1286152

**Published:** 2024-03-06

**Authors:** Daniel Marco Paredes-López, R. A. Robles-Huaynate, Marilu Roxana Soto-Vásquez, Rosa Amelia Perales-Camacho, Siever Miguel Morales-Cauti, Xiomara Beteta-Blas, Uriel Aldava-Pardave

**Affiliations:** ^1^Department of Animal Science, Universidad Nacional Agraria de la Selva, Tingo María, Peru; ^2^Faculty of Pharmacy, Universidad Nacional de Trujillo, Trujillo, Peru; ^3^Department of Animal and Public Health, Faculty of Veterinary Medicine, Universidad Nacional Mayor de San Marcos, Lima, Peru; ^4^Posgraduate School, Universidad Nacional Agraria de la Selva, Tingo María, Peru; ^5^Posgraduate School, Universidad Nacional Agraria La Molina, Lima, Peru

**Keywords:** *Piper aduncum*, *Artocarpus altilis*, *Morinda citrifolia*, intestinal health, performance indices

## Abstract

Bioactive plants such as *P. aduncum*, *M. citrifolia*, and *A. altilis* might improve intestinal health as an alternative to antibiotic growth promoters. The objective of this study was to determine the effect of the ethanolic extracts (EEs) of these plants on the intestinal health of broiler chickens. Cobb 500 chickens (*n* = 352) were distributed into eight treatments with four replicates and 11 chickens each. T1 received a base diet, and T2 received a base diet with 0.005% zinc bacitracin. T3, T5, and T7 were supplemented with 0.005% of *P. aduncum*, *M. citrifolia*, and *A. altilis* EE in the diet while T4, T6, and T8 with 0.01% of the extract. The EEs were supplemented with drinking water from 1 to 26 days of age. The following parameters were evaluated: hematological profiles at 28 days of age, blood metabolites profiles at 14, 21, and 28 days; *Escherichia coli*, *Staphylococcus aureus*, and *Lactobacillus* sp. abundance in the ileum mucosa and content at 21 and 28 days, and histomorphometry of the duodenum, jejunum, and ileum mucosa at 14, 21, and 28 d. Final weight (FW), weight gain (WG), feed intake (FI), and feed conversion rate (FCR) were evaluated at seven, 21, and 33 days of age. *M. citrifolia* and *A. altilis* EE at 0.01% increased blood glucose levels at 21 and 28 days of age, respectively, and *P. aduncum* and *M. citrifolia* EE at 0.01% increased triglycerides at 28 days of age; in addition, this EE did not have any effect on the AST and ALT profiles. The depths of the Lieberkühn crypts and the villi length to the crypt’s depth ratio increased with age on supplementation with 0.01% *M. citrifolia* and *A. altilis* EE at 21 days of age (*p* < 0.05). In addition, the depth of the crypts increased at 28 days of age (*p* < 0.05) in chickens supplemented with 0.01% *A. altilis* EE. The 0.01% *M. citrifolia* EE in diet decreased in the *Staphylococcus aureus* population in the ileal microbiota (*p* < 0.05). The FW and WG during the fattening and in the three stages overall increased, and the FCR decreased; however, the FI and the carcass yield did not change in the broiler chickens supplemented with 0.01% *M. citrifolia* EE (*p* < 0.05). Conclusively, the *M. citrifolia* EE at 0.01% of the diet improved intestinal health and thus the performance indices of the broiler chickens and did not have a detrimental effect on any of the parameters evaluated, so it is postulated as a potential alternative to AGP in poultry.

## Introduction

Since several years, antibiotics have been used as antimicrobial growth enhancers in animal feed to improve the productivity of various animal species and prevent the possible occurrence of diseases (1–5). However, the excessive use of antibiotics as growth enhancers in animal nutrition (6) has resulted in bacterial resistance in these animals (7, 8). Moreover, this has resulted in the presence of antibiotic residues in human food of animal origin (7, 9–12) and in the environment (13). Therefore, it is imperative to identify natural alternative products or additives that can replace antibiotics as preventive and growth-enhancing promoters (14–16).

Extracts or essential oils from different parts of plants, such as seeds, roots, and leaves, of medicinal, aromatic, flavoring, and other plants, are being increasingly used as phytogenic or phytobiotic additives, which function as growth enhancers (17–25).

The wide biodiversity of the Peruvian Amazon contains a diversity of native plants with nutraceutical properties, which potentially contain bioactive ingredients manifesting these properties. *Piper aduncum*, *Morinda citrifolia*, and *Artocarpus altilis* are found in the wild and domestic state in the Peruvian Amazon and scarcely used in traditional medicine by local populations.

However, these plants possess a variety of phytochemicals, such as phenolic, triterpene, flavonoid, and phenylpropanoid compounds, which in general possess antibacterial (26–28), antioxidant, and anti-inflammatory properties (29–32). In our previous study, it was found that *P. aduncum*, *M. citrifolia*, and *A. altilis* leaves contained 1,250 ± 0.06, 150.8 ± 0.06, and 224.3 ± 0.15 mg GAE/100 g of dried extract of polyphenols and 20.3 ± 0.10, 17.8 ± 0.10, and 30.7 ± 0.15 mg QE/100 g of dried extract of flavonoids, respectively. These plant bioactive compounds and activities might potentially improve the wellbeing, health, and productivity of animals. To our knowledge, this is the first study to demonstrate the effects of *P. aduncum*, *M. citrifolia*, and *A. altilis* EE on *in vivo* antimicrobial activity and intestinal histomorphometry.

Hence, this study aimed to determine the effects of the ethanolic extracts (EEs) of *P. aduncum*, *M. citrifolia*, and *A. altilis* leaves on the intestinal health of broiler chickens for improving productive performance indices.

## Materials and methods

### Leaves for ethanolic extract

The leaves of *P. aduncum*, *M. citrifolia*, and *A. altilis* were collected from fence plants, grown for edible and medicinal purpose in the Rupa-Rupa district of the Leoncio Prado Province in the Huánuco region of Peru. Harvesting was performed in the morning, and leaves that were neither very green nor too ripe were collected. The experiment involved the use of 5 kg of whole fresh leaf in well-conserved conditions. These were dried at 60°C in a forced ventilation stove (Memmert, UN110 plus, Germany) for 72 h, subsequently ground using a 1 mm diameter sieve in a grinder (Thomas Willey, United States), and stored in dark using tightly sealed recipients. This procedure was adapted from Lal et al. (33).

To obtain the EEs of *P. aduncum*, *M. citrifolia*, and *A. altilis* leaves, 50 g of leaf powder from each of the plants was collected in a cartridge and placed in a stove at 40°C. This was then placed in a Soxhlet extractor, and extraction was performed by placing 150 mL of 70% ethanol in an Erlenmeyer flask, where the volume was equivalent to three times the weight of the leaf powder. The EEs were dehydrated in a rotary evaporator (Heidolph, Germany) at 40°C with reduced pressure to eliminate all the solvent and then were completely dried. These dried EEs were weighted, and each 10 g was reconstituted with tween:water (80:20 mL) to obtain a10% solution, which was used to calculate the 0.005 and 0.01% EEs in the chicken diet. The EEs obtained from *P. aduncum*, *M. citrifolia*, and *A. altilis* leaves were stored in amber jars and subjected to preliminary phytochemical screening (Table 1).

**Table 1:** Phytochemical screening of ethanolic extracts of *P. aduncum*, *M. citrifolia*, and *A. altilis* leaves.

Metabolite	Test	*P. aduncum*	*A. altilis*	*M. citrifolia*
Alkaloids	Dragendorff	++	++	−
	Mayer	++	++	−
	Wagner	++	++	−
Lactones	Baljet	−	+	−
Phenolic compounds	Cloruro férrico	+++	+++	−
Flavonoids	Shinoda	+++	++	+
Antocianidins	Antocianidina	+	+	−
Catequins	Catequinas	+	+	−
Triterpens and Esteroids	Liebermann–Burchard	++	+	+
Cardenólids	Kedde	−	−	−
Quinones	Bornträger	+	++	+
Saponins	Foam	+	−	−
Resins	Resins	+	−	−
Reducing sugars	Fehling	++	−	+
Aminoácids	Ninhidrina	+	+	+

### Rearing the broiler chickens

This study involving animals was reviewed and approved with authorization No 2021-5 by the Ethics and Animal Wellbeing Committee from the Faculty of Veterinary Medicine, Universidad Nacional Mayor de San Marcos. The location of this study was at 09° 17′ 58″ south latitude and 76° 01′ 07″ west longitude, at an altitude of 660 m.a.s.l., an annual pluvial precipitation of 3,293 mm, an average annual temperature of 24.85°C, and relative humidity of 80% (34).

A 20 m long × 10 m width shed was used, in which 33 metal cages 82 cm width, 1.28 cm depth and 70 cm height were installed. Each cage was equipped with a 100 watt light bulb, a conical feeder, a drinker, and a 10 cm-high wood shaving bed. The temperature and minimum and maximum humidity were determined using a temperature and humidity reader. The average temperature and relative humidity of the shed during the experimental rearing were 28.3°C and 82.6%, respectively.

A total of 352 1 day-old Cobb 500 weighing 40 ± g were reared. The chickens were divided into eight treatment groups, with each treatment having four replicates and 11 chickens each, placed in 32 separate cages. All birds received the same handling and feeding conditions, comprising a base diet during the initial (1–7 days), growth (8–21 days), and finishing (22–33 days) stages.

### Experimental diets and feeding

The chicken diets were formulated in the Mixit-2 program, based on the information by Rostagno et al. (35). First, a premix of the micronutrients with raw insoluble fiber was prepared to efficient homogenization in the diet, and mixing of the components was performed in a horizontal mixer for 10 min (Table 2). This diet was fed to chickens as powder at an average daily dose per chicken of 26.3 g, 76.85 g, and 144.43 g for the initial, growing, and fattening stages, respectively. The nutritional compositions of the initial, growth, and finishing stages (1–33 days old) were determined according to the requirements for each stage (19). For this purpose, samples of base diet and with ZB for each broiler chicken phase were sent to the laboratory of nutrition from the Department of Animal Science, Universidad Nacional Agraria de la Selva for dry matter (DM) and chemical analysis. To determine DM content, the samples were dried in an air-forced oven (Memmert, UN110 plus, Germany) at 105°C for 4 h. The samples were analyzed for ashes after 12 h of combustion in a muffle furnace at 600°C (Linn Electro Therm, LM-312.06, Germany); crude protein (CP) using a Kjeldahl nitrogen analyzer (Buchi digest automatic, K-438, and Buchi distillation unit K-350, Switzerland); ethereal extract using an extractor (Ankom XT10, United States); total fiber was determined by a semiautomatic fiber analyzer Ankom 200, USA. The nitrogen-free extract was calculated by the difference between DM and the nutrients determined in the proximal analysis of the diets. The chemical analysis of the diets is shown in Table 3.

**Table 2:** Experimental diets formulated for male broiler chickens for the initial (1–7 days old), growth (8–21 days old), and fattening (22–33 days old) stages.

Ingredients (%)	Initial			Growth			Fattening		
	T1	T2	T3–T8	T1	T2	T3–T8	T1	T2	T3–T8
Corn	52.8	51.2	53.96	51.2	51.2	51.2	53.96	53.96	53.96
Palm oil	2.62	4.46	5.5	4.46	4.46	4.46	5.5	5.5	5.5
Soybean cake (46%)	36.4	39.9	36.37	39.9	39.9	39.88	36.37	36.37	36.37
Calcium carbonate	0.89	0.79	0.75	0.79	0.79	0.79	0.75	0.75	0.75
Dicalcium phosphate	0.21	1.8	1.58	1.8	1.8	1.8	1.58	1.58	1.58
Salt	0.23	0.22	0.2	0.22	0.22	0.22	0.2	0.2	0.2
Premix Vit + Min.	0.15	0.15	0.1	0.15	0.15	0.15	0.1	0.1	0.1
Aflaban	0.05	0.05	0.05	0.05	0.05	0.05	0.05	0.05	0.05
Coccidiostat	0.05	0.05	0.05	0.05	0.05	0.05	0.05	0.05	0.05
Butylated hydroxytoluene	0.05	0.05	0.05	0.05	0.05	0.05	0.05	0.05	0.05
Choline chloride	0.25	0.2	0.2	0.2	0.2	0.2	0.2	0.2	0.2
Sodium butyrate	0.1	0.1	0.1	0.1	0.1	0.1	0.1	0.1	0.1
Sodium bicarbonate	0.46	0.45	0.44	0.45	0.45	0.45	0.44	0.44	0.44
Lysine (78.4%)	0.31	0.22	0.24	0.22	0.22	0.22	0.24	0.24	0.24
Methionine (99%)	0.25	0.23	0.2	0.23	0.23	0.23	0.22	0.22	0.22
Threonine (98%)	0.11	0.09	0.09	0.09	0.09	0.09	0.09	0.09	0.09
Valine (99%)	0.09	0.06	0.06	0.06	0.06	0.06	0.06	0.06	0.06
BMD (10%)	0	0.01	0	0	0.1	0	0	0.05*	0
Extruded soybean	5	0	0	0	0	0	0	0	0
Oxytetracycline (99%)	0	0	0	0	0	0	0	0	0
Total	100	100	100	100	100	100	100	100	100

T1, negative control; T2, positive control; T3–T8, supplemented with ethanolic extract up to 26 days of age.

**Table 3:** The nutritional composition of the experimental diets for male broiler chickens during the initial, growth, and fattening stages (1–33 d old).

Diet samples	Treatments	Dry matter (DM) (%)	Ash (% of DM)	Crude protein (% of DM)	Extracto etereo (% of DM)	Total fiber (% of DM)	ELN (% of DM)
Initial base	T1, T3, T5, T6, T7, T8	90.10	7.12	23.5	5.23	2.43	52. 27
Initial with ZB	T2	90.05	7.10	23,15	5.19	2.49	52.12
Growth base	T1, T3, T5, T6, T7, T8	91.24	6.81	22.13	7.02	2.32	52.96
Growth with ZB	T2	91.52	6.86	22.04	7.16	2.35	53.11
Fattening base	T1, T3, T5, T6, T7, T8	88.72	6.12	20.34	7.92	2.40	51.91
Fattening with ZB	T2	89.05	6.11	20.41	7.91	2.46	52.14

The diet provided in this study was carefully monitored to ensure that aflatoxin levels were well below the established safety limits for animal feed. This precautionary measure was taken to safeguard the animals’ health and welfare. Aflatoxin contamination in animal feed can pose serious health risks, including impaired growth and liver damage (36). By maintaining feed quality within safe limits and adding plant products (32, 37, 38), we aimed to minimize any potential influence of aflatoxins on the study results.

### Extract supplementation

*P. aduncum*, *M. citrifolia*, and *A. altilis* EE at 0.005 and 0.01% of the diets were calculated and supplemented with the drinking water daily in plastic cylindrical 2 L volume and manual handling drinkers from 1 to 26 days of age. The average volume of water supplied for each chicken was 65.75, 192.5, and 361.10 mL for the initial, growing, and fattening stages, respectively. The extracts from the three plants were formulated at a concentration of 100 mg/mL in tween:water solution. At this concentration, the solution was separated into aliquots at the beginning of the experiment, according to the calculations at 0.005 and 0.01% of the weight of the diet obtained for each day of the experiment. The aliquots were frozen at −10°C to allow removal out of a single aliquot daily for the volume that corresponded to each day for the total experimental chickens. The total intake of the EE was 4.27 g for each of the 0.005% supplement groups and 8.55 g for each of the 0.01% supplemented groups of chicken, for the *P. aduncum*, *M. citrifolia* and *A. altilis* EE, respectively.

### Blood samples, hematology, and blood metabolite profiles

Blood samples were collected by puncturing the jugular vein. Blood samples to generate hematological profiles were obtained in 2 mL vacutainers containing 2 mg heparin. Blood samples for metabolite profiles were collected in 4 mL vacutainers, which, once coagulated, were centrifuged at 1500 rpm for 5 min. Subsequently, the serum was separated into 2 mL Eppendorf tubes and stored at −10°C until its spectrophotometric analysis. Thirty-three chickens were sampled at 28 days of age for their hematological profiles and at 14, 21, and 28 days of age for their blood metabolite profiles.

Whole blood was used to determine the erythrocyte count, total and differential leukocytes, hematocrit using the microhematocrit method, and hemoglobin levels using the cyanmethemoglobin method. Simultaneously, these data were used to obtain the indices for mean corpuscular volume (MCV), mean corpuscular hemoglobin (MCH), and mean corpuscular hemoglobin concentration (MCHC) (39).

Serum glucose profiles were determined by the glucose oxidase/peroxidase method; total protein contents were determined using the EDTA-Cu complex in sodium hydroxide method; and albumin levels were determined using the bromocresol green method (40, 41). Similarly, the total cholesterol, alanine transaminase (ALT), and aspartate transaminase (AST) levels were determined using specific kits (Laboratorios QAC, Spain). Optical density measurements were performed at 515 and 530 nm using an Auto Chemistry Analyzer-AS 830 spectrophotometers (Italy).

### Intestinal content samples and microbiological culture

Three chickens were randomly selected from each of the eight treatment groups at 21 and 28 d of age and euthanized by breaking the atlanto-occipital joint. The ileum was immediately dissected, approximately 30 cm long after the Meckel’s diverticulum, toward the cecum (42, 43). From the opened ileum, one gram of intestinal content, including scrapes of the mucosa, was obtained and placed in a sterile Petri dish.

Colonies of the broiler chicken microbiota, such as, *Escherichia coli*, *Lactobacillus* sp., and *Staphylococcus* sp., (42), were cultivated to serve as marker for evaluating the *in vivo* antimicrobial activity of the EE. *Escherichia coli*, *Lactobacillus* sp., and *Staphylococcus aureus* were cultivated on MacConkey, MRS, and salty Mannitol agar (Merck, Darmstadt, Germany). The plates were incubated for 24 h at 37°C. Bacterial counts were measured as the number of colonies forming units (CFUs) per gram of ileum content and expressed as logarithm base 10 of these CFUs (42, 43).

### Intestinal tissue samples and evaluation of intestinal morphometry

Four chickens were randomly selected from each of the eight treatment groups at 14, 21, and 28 days of age and euthanized by breaking the atlanto-occipital joint. Their digestive tracts were immediately dissected, and an approximately 5 cm segment was taken from the middle of each of the following sections: the duodenum, jejunum, and ileum (42, 43), which were opened lengthwise and transversely sectioned.

Tissues were fixed by submerging them in a 3–4-fold sterile physiological solution to detach the intestinal contents from the mucosa and later stapled to a thick cardboard base to hold the segments straight. The three segments from each bird were placed in 100 mL of a 10% formaldehyde solution in physiological solution. The intestinal samples were processed using conventional histological methods and stained using hematoxylin and eosin (44).

**Figure 1:**
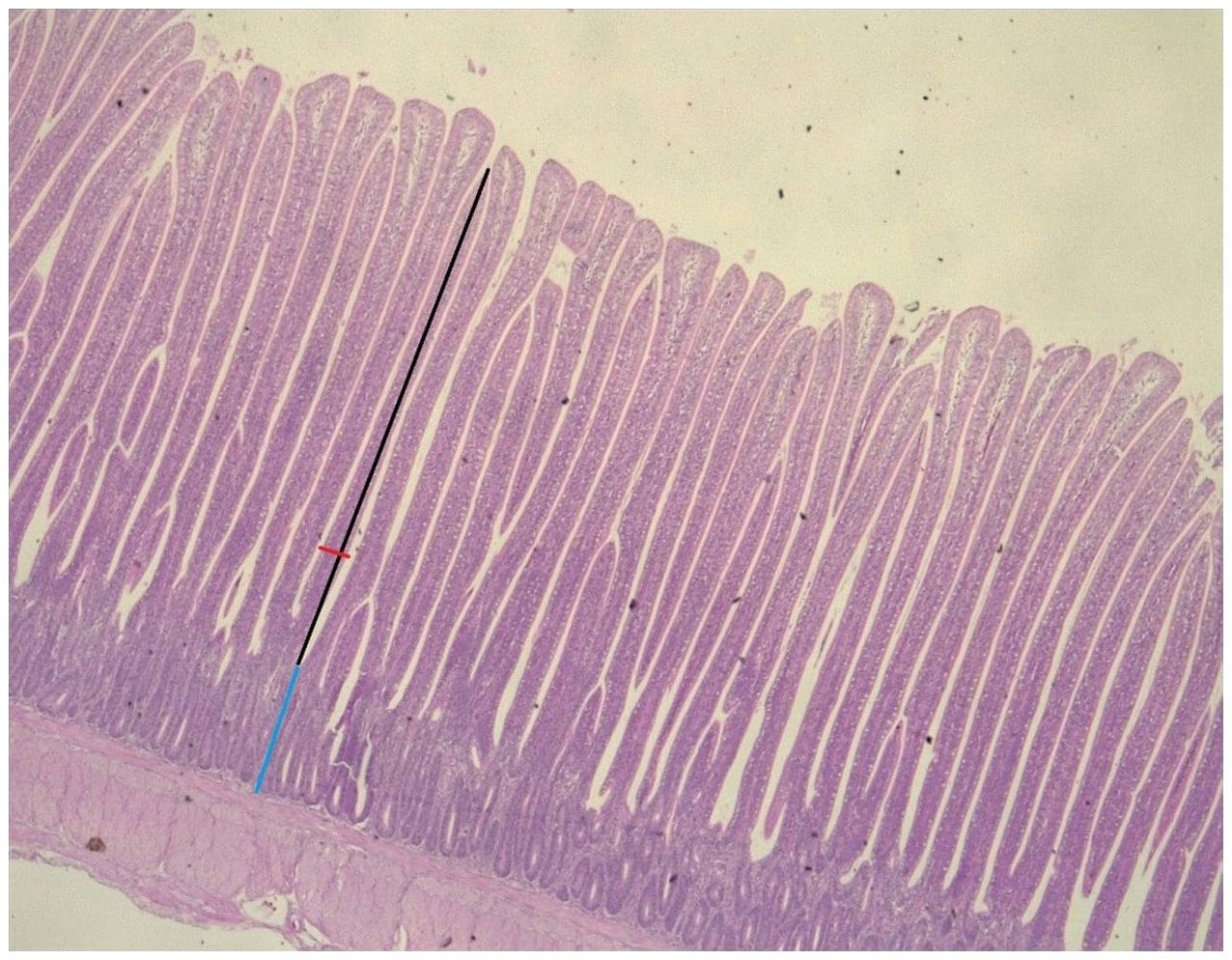
Villous height (VH), villous width (VW), and crypt depth (CD) determination in the jejunum of broiler chicken at 1 Ox. VH, black arrow; VW, red arrow; CD, blue arrow (hematoxylin and eosin staining).

A DM 750 optical microscope with a digital camera (ICC50) and a LAS 4.12 EZ software (Leica, Germany) was used. The system allows measurements of the distance between any pair of user-defined fixed points. The villus length was measured from the top to the apex of the Lieberkühn crypt entrance. The width of the villi was measured as a perpendicular line to the center of the villi. The depth of the Lieberkühn crypt was measured from its entrance to the base zone (Figure 1). The length and width of the intestinal villi and depth of the crypts were determined by measuring ten villi at 10×; the averages of every intestinal segment corresponding to each animal were obtained and registered in microns (μm).

### Determining the productive parameters

To determine the effect of different levels of *P. aduncum*, *M. citrifolia*, and *A. altilis* EE on the productive performance of broiler chickens, the feed consumed and leftover daily by all experimental chickens were recorded during the 35 days of the experiment. The body weights of all experimental broiler chickens were recorded at 7, 21, and 35 days of age. Using these data and adapting the conventional productive performance ratios for animal production (45), the following ratios were calculated:

- Daily feed intake (DFI): feed was weighed and provided to each replicate; later, the leftovers were deducted and divided between the number of chickens and days in the stage. This was calculated in the following manner:

$$DFI\left(g\right)=\frac{\mathrm{Total}\;\mathrm{weight}\;to\;\mathrm{feed}\;\mathrm{intake}}{\mathrm{Number}\;\mathrm{of}\;\mathrm{days}\;\mathrm{of}\;\mathrm{breeding}}$$

- Carcass yield (CY): it was calculated using the relationship between the weight without disposal and the live weight in the lot. The following formula was used for calculations:

$$CY\left(\%\right)=100\times \frac{\mathrm{Weight}\;\mathrm{of}\;\mathrm{the}\;\mathrm{lot}\;\mathrm{without}\;\mathrm{disposals}}{\mathrm{Live}\;\mathrm{Weight}\;\mathrm{of}\;\mathrm{the}\;\mathrm{lot}}$$

- Daily weight gain (DWG): chickens were weighed at 6:00 a.m. before the feed was provided. The calculations were performed using the following formula:

$$DWG\left(g\right)=\frac{\mathrm{final}\;\mathrm{weight}-\mathrm{initial}\;\mathrm{weight}}{\mathrm{time}\;\left(\mathrm{days}\right)}$$

- Cumulative weight gain (CWG): it was calculated as the relationship between the final weight minus the initial weight of the lot and the number of finished birds in the lot. The calculations were performed as follows:

$$CWG\left(g\right)=\frac{\mathrm{final}\;\mathrm{lot}\;\mathrm{weight}-\mathrm{initial}\;\mathrm{lot}\;\mathrm{weight}}{\mathrm{number}\;\mathrm{of}\;\mathrm{finished}\;\mathrm{birds}\;\mathrm{in}\;\mathrm{the}\;\mathrm{lot}}$$

- Feed conversion rate (FCR): it was calculated using the relationship between total feed consumption and weight gain. The following formula was used to calculate this:

$$FCR=\frac{\mathrm{Total}\;\mathrm{feed}\;\mathrm{consumption}}{\mathrm{Total}\;\mathrm{weight}\;\mathrm{gain}}$$

### Statistical analyses

To evaluate the effect of the EE supplementation on the variables under study in relation to the chicken age, the data on blood metabolite profiles, development of the villi, and Lieberkühn crypts in the intestinal segments were processed by means of a general factorial design with three ages of chickens, six EE levels +2 controls, and for bacterial count, two ages of chickens. The guidance for statistics analysis was taken from Bashir, et al., (46) and Pollesel et al., (47). Data of hematology and performance indices were submitted to a completely randomized design with eight treatments, four replicates with eight chickens each. Data for the length and width of the villi, depth of the crypts, bacterial count, and some data of hematology and performance indices were firstly transformed using the square root, Box-Cox, or base 10 logarithms and then tested for normality and homoscedasticity with the Shapiro–Wilk and Levene test, respectively. One-way analysis of variance (ANOVA) was used to test the effect of EEs on the hematological and performance parameters, and two-way ANOVA procedure was employed to test the effect of EEs on metabolites profiles, bacterial count, and intestinal histomorphometry. Significant differences were declared for *p* ≤ 0.05. The Student–Newman–Keuls (SNK) multiple comparison test was used for comparison between treatments and ages. The Infostat statistical software was used for data processing (48).

## Results

### Hematological and blood metabolites profiles

The erythrocyte, hematocrit, and hemoglobin profiles; MCV, MCH, and MCHC indices; and total leukocyte, lymphocyte, and granulocyte counts of the broiler chickens are shown in Table 4. The granulocyte counts in chickens supplemented with 0.01% *A. altilis* EE increased in relation to supplementation with the same concentration of *P. aduncum* EE (*p* < 0.05). However, this and the other hematological profiles of the chickens supplemented with *A. altilis* EE were similar (*p* > 0.05) to those obtained from the chickens in the control groups, and those supplemented with 0.005 and 0.01% *P. aduncum* and *M. citrifolia* EE.

**Table 4:** Variance analysis of erythrocyte and leukocyte profiles of broiler chickens supplemented with *P aduncum*, *M. citrifolia*, and *A. altilis* EE at 0.005 and 0.01% of the diet.

Treatments EE level (%)		Hematological profiles								
		HTO (%)	HB (mgdL^−1^)	ERY (x10^6^ μL^−1^)	MCV (fL)	MCH (pg)	MCHC (gdL^−1^)	*LINF (%)	LEU (×10^−3^ μL^−1^)	**GRA (%)
Control	−	30.75	10.15	3.48	88.47	29.20	33.01	62.75	19.67	37.25ab
	+	29.25	9.65	3.33	87.91	29.00	32.99	67.25	17.32	32.75ab
*P. aduncum*	0.005	28.25	9.33	3.23	87.53	28.90	33.02	64.50	9.95	35.50ab
	0.01	27.00	8.88	3.10	87.05	28.62	32.87	67.75	13.23	29.75b
*M. citrifolia*	0.005	27.00	8.83	3.10	87.02	28.47	32.71	69.50	14.53	30.75ab
	0.01	29.50	9.60	3.33	87.97	28.78	32.71	61.25	14.40	37.25ab
*A. altilis*	0.005	28.50	9.40	3.25	87.64	28.90	32.98	63.00	12.72	34.50ab
	0.01	29.50	9.75	3.35	88.06	29.10	33.05	59.00	19.68	41.00a
*Variance analysis*										
*p*-value		0.2394	0.2181	0.2470	0.2321	0.1158	0.3303	0.1722	0.1470	0.0374
CV (%)		7.56	7.67	6.63	0.94	1.24	0.76	8.66	34.40	0.02
Aj. *R*^2^ (%)		8.84	9.99	8.38	9.30	16.70	4.80	12.60	14.30	26.70

^*^Different letters denote significant differences, NSK test (*p* < 0.05). The residuals did not meet the assumptions of normality and homoscedasticity; ^**^the Box-Cox transformation with lambda = −2 was done. Different letters indicate significant differences (*p* < 0.05).HTO, hematocrit; HB, hemoglobin; ERY, erythrocytes; MCV, mean cell volume; MCH, mean corpuscular hemoglobin; MCHC, mean corpuscular hemoglobin concentration; LINF, lymphocytes; LEU, leucocytes; GRA, granulocytes.

The levels of glucose, triglycerides, AST, ALT, total protein, albumin, and globulin profiles, which are important markers for animal physiology, were evaluated. Table 5 presents these blood metabolites on supplementation with *P. aduncum*, *M. citrifolia*, and *A. altilis* EE.

**Table 5:** Variance analysis of blood metabolites profiles of broiler chickens supplemented with *P. aduncum*, *M. citrifolia*, and *A. altilis* EE.

Treatments	EE level (%)	GLUC (mmol/L)	TRIG (mg/dL)*	AST (UI/L)**	ALT (UI/L)	TP (g/dL)	ALB (g/dL)**	GLOB (g/dL)
Control	−	233.67	61.68a	210.45	17.5	2.33	1.33	0.98
	+	213.58	49.58ab	210.86	18.00	2.19	1.26	0.94
*P. aduncum*	0.005	219.17	40.57b	220.31	18.75	2.32	1.33	0.98
	0.01	224.67	45.37ab	207.26	17.67	2.24	1.31	0.92
*M. citrifolia*	0.005	222.50	46.52ab	202.16	17.5	2.30	1.35	0.96
	0.01	234.50	45.83ab	213.94	18.08	2.36	1.35	1.00
*A. altilis*	0.005	216.58	45.96ab	191.25	18.42	2.32	1.40	0.91
	0.01	222.58	41.45b	220.85	17.75	2.22	1.33	0.88
Ages (Days)	14 days	196.72b	38.60c	187.36b	11.00b	1.96b	1.23b	0.73b
	21 days	239.88a	46.33b	215.58a	21.16a	2.41a	1.36a	1.04a
	28 days	233.63a	57.59a	227.43a	21.72a	2.48a	1.42a	1.06a
*p-value*								
Treatment (T)		0.4884	0.0001	0.256	0.9744	0.44	0.7	0.727
Age (A)		0.0001	0.0325	0.0001	0,0001	0,0001	0,0001	0.0001
^1^T × A		0.0053	0.0142	0.456	0.0779	0.174	0.103	0.505
^2^VC. (%)		12.03	3.23	2.66	17.7	8.96	42.91	18.23
*R*^2^		99.8	52.39	43.31	78.03	67.43	41.33	55.44
Adjusted *R*^2^		41.42	37.18	25.21	71.01	57.03	22.58	41.21

Different letters denote significant differences (*p* < 0.05).GLU, glucose; TRIG, triglycerides; AST, aspartate transaminase; ALT, alanine; transaminase; TP, total protein; ALB, albumin; GLOB, globulin; transformation Box-Cox with lambda = −0.3838 for TRIG (*), logarithm base 10 for TGO (**) and ALB (**).

*M. citrifolia* and *A. altilis* EE at 0.01% increased blood glucose levels at 21 and 28 days of age, respectively, compared with the levels obtained at 14 days of age (*p* < 0.05) (Tables 5, 6). Similar results were observed for increased triglyceride levels using *P. aduncum* and *M. citrifolia* EE at 0.01%, for which the triglycerides increased at 28 days of age, compared with those obtained at 14 days of age (*p* < 0.05) (Tables 5, 7).

**Table 6:** Variation of glucose levels with chicken age on supplementation with *P. aduncum*, *M. citrifolia*, and *A. altilis* EE.

Treatments	EE levels (%)	14 days	21 days	28 days
Control	−	241.75a	243.75	216.50
	+	172.75b	234.00	234.00
*P. aduncum*	0.005	191.50	234.00	232.00
	0.01	188.25	258.00	227.75
*M. citrifolia*	0.005	200.50	249.25	217.75
	0.01	199.25B	270.00aA	234.25
*A. altilis*	0.005	200.75	198,50b	250.50
	0.01	179.00B	232.5	256.25A

Different letters denote significant differences (*p* < 0.05). Lowercase letters between the treatments within each age (column). Uppercase letters between the ages within each treatment (row).

**Table 7:** Variation of triglyceride profiles with chicken age on supplementation with *P. aduncum*, *M. citrifolia*, and *A. altilis* EE.

Treatment	EE levels (%)	14 days	21 days	28 days
Control	−	45.25	64.32	83.61
	+	50.84	39.82	61.29
*P. aduncum*	0.005	30.92	42.59	52.06
	0.01	29.28B	51.25	66.68A
*M. citrifolia*	0.005	40.96	38.98	64.94
	0.01	31.70B	46.11	69.93A
*A. altilis*	0.005	45.34	50.98	42.14
	0.01	42.59	42.95	38.97

Different letters denote statistical differences (*p* < 0.05): lowercase letters between the treatments within the same age (column), and uppercase letters between the ages within each treatment (row).

### Intestinal microbiology

The microbiological population obtained from the content and mucosa of the ileum from broiler chickens at 21 and 28 days of age as log10CFU/g of fresh intestinal content is shown in Table 8. The abundance of *Staphylococcus aureus* as (log10CFU) in the ileum of the broiler chickens decreased on dietary supplementation with 0.01% *M. citrifolia* EE (*p* < 0.05), in comparison with the abundance in negative control group and on supplementation with 0.005% *P. aduncum* and *A. altilis* EE. However, there was no effect of the EE from these three plants on the populations (log10CFU) of *E. coli* and *Lactobacillus* sp. in the ileum of broiler chickens (*p* > 0.05) compared with that obtained from the chickens in the control group (Table 8).

**Table 8:** Bacterial abundance in ileal mucosa of broiler chickens supplemented with *P. aduncum*, *M. citrifolia*, and *A. altilis* EE.

Treatments	EE level (%)	*Staphylococcus* sp. (Log_10_ CFU/mL)	*E. coli* (Log_10_ CFU/mL)	*Lactobacillus* sp. (Log_10_ CFU/mL)
Control	Control +	5.77ab	4.42	6.72
	Control −	6.64a	5.49	6.46
*P. aduncum*	0.005	6.67a	5.63	7.53
	0.01	6.14ab	5.34	6.81
*M. citrifolia*	0.005	6.07ab	6.45	6.73
	0.01	4.46b	5.11	6.69
*A. altilis*	0.005	6.68a	6.09	6.63
	0.01	5.97ab	5.11	6.78
*Age*				
21 days		6.11	6.17 A	6.72
28 days		5.99	4.74 B	6.86
*p*-value: Treatment (T)		0.0376	0.2692	0.2767
Age (A)		0.7199	0.0007	0.4744
T*A		0.4851	0.5591	0.3689
CV (%)		18.79	24.35	9.94
*R*^2^		42.96	47.7	35.51
Adjusted *R*^2^		16.23	23.18	5.28

Different letters between rows denote significant differences for the SNK test at 5%. The base 10 logarithmic transformation is used for the three variables.

### Intestinal morphometry

The length and width of the intestinal villi, the depth of the Lieberkühn glands, and villi length to Lieberkühn crypt depth ratio were evaluated for duodenal, jejunal, and ileal segments. These structures are important markers of the pathophysiological anatomy of the small intestine in different animal species. Table 9 presents the results from measuring these structures in broiler chickens on supplementation with 0.005 and 0.01% *P. aduncum*, *M. citrifolia*, and *A. altilis* EE in drinking water.

**Table 9:** Variance analysis and morphometry of the mucosa from the duodenum, jejunum, and ileum of broiler chickens supplemented with *M. citrifolia*, *P. aduncum*, and *A. altilis* EE.

Factors	***Villi length (VL) (μm)	**Crypt depth (CD) (μm)	*Villi width (VW) (μm)	****VL/CD	
Age (A)	0.0000	0.0000	0.0337	0.0001	
Treatment (T)	0.1304	0.0164	0.0394	0.0017	
Segment (S)	0.0000	0.0000	0.0095	0.0001	
A*T	0.0021	0.0000	0.0313	0.0001	
E*S	0.0000	0.0280	0.2611	0.0012	
T*S	0.6906	0.8131	0.8659	0.3811	
E*T*S	0.1650	0.9276	0.8404	0.0604	
VC (%)	6.95	2.83	0.11	12.20	
Adjusted *R*^2^ (%)	87.18	35.60	6.71	83.14	
*Treatments*					
Control	−	1080.36	194.98	122.77	5.48^b^
	+	1157.00	187.14^b^	121.34	6.12^a^
*P. aduncum*	0.005	1079.59	192.87	125.42	5.53^b^
	0.01	1115.79	196.55	120.45^b^	5.65^b^
*M. citrifolia*	0.005	1157.32	203.60	123.99	5.59^b^
	0.01	1147.14	213.05^a^	121.70	5.33^b^
*A. altilis*	0.005	1090.34	201.75	129.23^a^	5.35^b^
	0.01	1111.28	202.56	123.42	5.44^b^
*Age*					
14 days	1009.23^c^	186.16^b^	121.47^b^	5.36^b^	
21 days	1201.73^a^	223.64^a^	123.37^ab^	5.35^b^	
28 days	1144.92^b^	189.08^b^	125.66^a^	5.98^a^	
*Segment*					
Duodenum	1684.00^a^	209.14^a^	125.94^a^	8.07^a^	
Jejunum	1049.65^b^	203.07^a^	121.03^b^	5.16^b^	
Ileum	720.17^c^	185.36^b^	123.56^ab^	3.87^c^	

Different letters denote significant differences for the SNK test (*p* < 0.05). The Box-Cox transformation was done with lambda = 2 (*), base 10 logarithm (**), square root (***), and lambda = 0.4646465.

In this study, the Lieberkühn crypt depth increased with age in chickens on supplementation with *M. citrifolia* and *P. aduncum* EE at 0.01%, compared with those in the negative and positive control of 21 days-olds (*p* < 0.05) (Tables 9, 10). In addition, chickens supplemented with 0.01% *A. altilis* EE showed a crypt depth increase compared with that obtained in the positive control at 28 days of age (*p* < 0.05) (Tables 9, 10).

**Table 10:** Variation of Lieberkühn crypts depth with broiler chickens age on supplementation with *M. citrifolia*, *P. aduncum*, and *A. altilis* EE.

Treatment	Extract dose	Chicken age (Days)		
		14	21	28
Control	−	192.12A	194.45bA	198.43A
	+	197.31A	199.28bA	166.69bA
*P. aduncum*	0.05	177.18B	237.67A	170.38B
	0.01	178.81B	247.43aA	171.63B
*M. citrifolia*	0.05	188.09A	229.13A	195.82A
	0.01	196.53B	244.36aA	201.38B
*A. altilis*	0.05	179.48B	223.56A	204.65
	0.01	181.00A	219.37A	209.32aA

Different letters denote significant differences, SNK test (*p* < 0.05). Lowercase letters denote significance between treatments within each age (column). Uppercase letters denote significance between each treatment (row).

**Figure 2:**
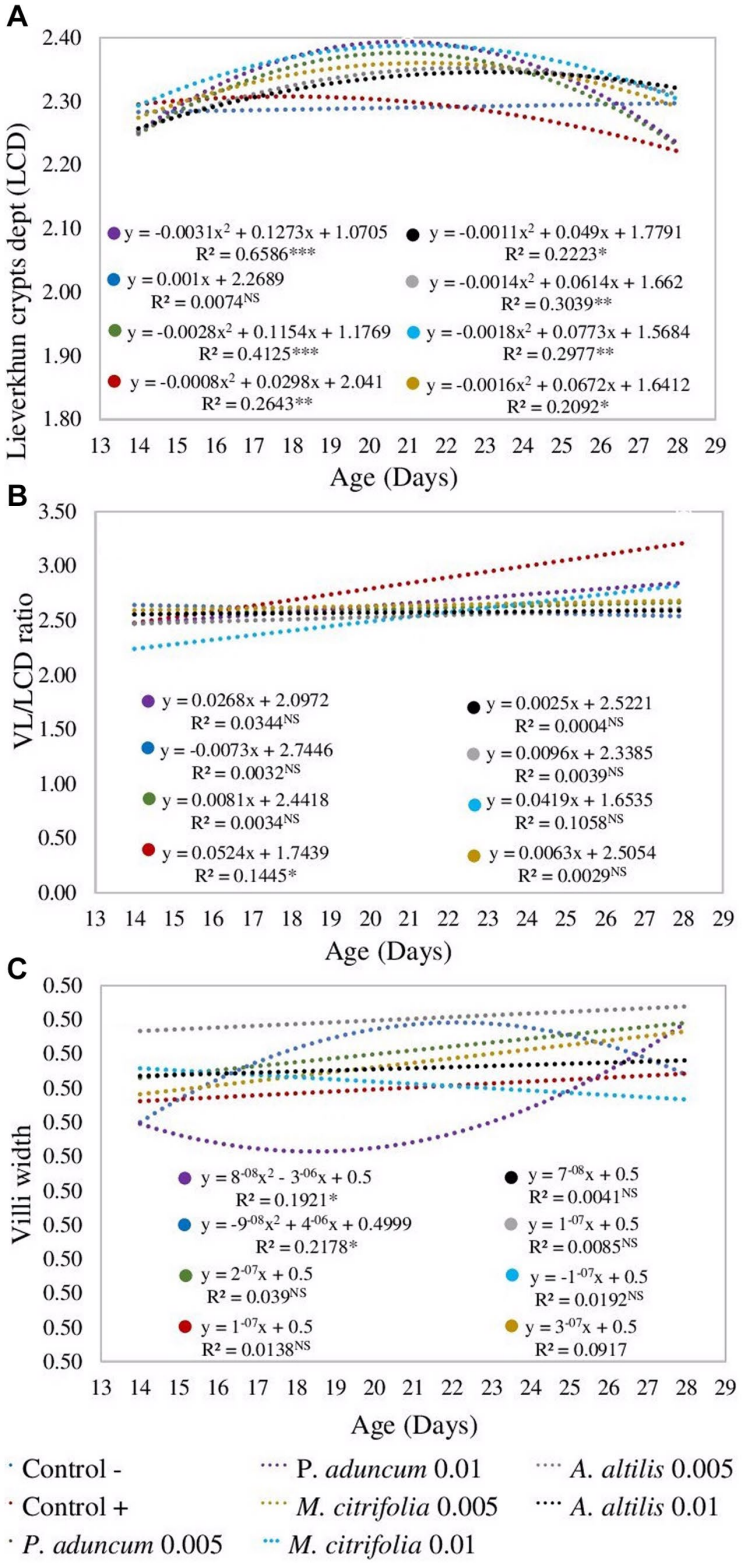
Regression analysis from the effect of the broiler chicken age on the crypt depth **(A)**, villi width **(C)**, and villi length to Lieberkühn crypt depth ratio **(B)** on supplementation 0.005 and 0.01% *P. aduncum*, *M. citrifolia*, and *A. altilis* EE. *value of *p* <0.05, **value of *p* <0.01, and ***value of *p* <0.001. **(A)** Data were transformed with logarithm base 10, **(B)** with Box-Cox = 46/99, and **(C)** with Box-Cox = 2. VL, villi length.

Additionally, in all studied supplementations, crypt depth was influenced by chicken age at the two evaluated EE concentrations (*p* < 0.05); however, in the negative control group, it was not dependent on the chicken age (*p* > 0.05) (Figure 2A). Moreover, villus width increased in the group of chickens supplemented with 0.005% *A. altilis* EE, compared with that in the positive control group, and in those supplemented with 0.01% *P. aduncum* EE at 21 d of age (*p* < 0.05) (Tables 9, 11). However, the villus width increased in a quadratic trend with chicken age on supplementation with 0.01% *P. aduncum* EE and decreased in a quadratic trend with age in the negative control group (*p* < 0.05) (Figure 2C).

**Table 11:** Variation of villi width with broiler chickens age on supplementation with *M. citrifolia*, *P. aduncum*, and *A. altilis* EE.

Treatment	Extract level	Chickens age (Days)		
		14	21	28
Control	−	117.85	128.52	122.66
	+	121.43	118.38b	124.44
*P. aduncum*	0.05	123.89	122.28	130.55
	0.01	117.71	116.00b	128.81
*M. citrifolia*	0.05	120.43	124.43	127.42
	0.01	122.35	123.78	119.12
*A. altilis*	0.05	124.34	137.18a	127.19
	0.01	124.41	119.89	126.22

Different letters denote significant differences SNK (*p* < 0.05). Lowercase letters denote significance between treatments within each age (column). Uppercase letters denote significance between ages within the same treatment (row).

Furthermore, the villus length and the Lieberkühn crypt depth ratio for the chickens supplemented with 0.01% *M. citrifolia* EE and the positive control increased at 21 days of age, in comparison with those obtained at 14 days of age (*p* < 0.05). These parameters were similar to the two groups (*p* > 0.05), and greater than those obtained for chickens supplemented with *P. aduncum* and *A. altilis* EE, and for the chickens from the negative control group (*p* < 0.05) (Tables 9, 12). However, the villus length and Lieberkühn crypt depth ratio was independent of chicken age on supplementation with *M. citrifolia*, *P. aduncum*, and *A. altilis* EE (*p* > 0.05) (Figure 2B).

**Table 12:** Variation of villi length to Lieberkühn crypt depth ratio (VL/LCD) with broiler chicken age on supplementation with *M. citrifolia*, *P. aduncum*, and *A. altilis* EE.

Treatment	EE level (%)	14 Days	21 Days	28 Days
Control	−	5.52	5.66	5.26b
	+	5.13B	6.27a	7.06aA
*P. aduncum*	0.005	5.56	5.18	5.85b
	0.01	5.41	5.20B	6.38A
*M. citrifolia*	0.005	5.82	4.94b	6.05b
	0.01	4.72B	5.16	6.17A
*A. altilis*	0.005	5.26	5.21	5.59b
	0.01	5.49	5.25	5.57b

Different letters denote significant differences, SNK test (*p* < 0.05). Lowercase letters compare the LV/PC between the treatments within the same age (column). Uppercase letters compare the LV/PC between the ages within the same treatment (row).

Additionally, this interaction increased the length of the villi for the chickens at 21 days of age, compared with those at 14 days of age (*p* < 0.05), but this pattern was similar to that obtained for the villi of chickens from the negative control group (*p* > 0.05) (Table 9).

### Productive indices

The total weight, weight gain, feed conversion rate, and feed intake of the broiler chickens were evaluated at each of the following stages: initial, growth, and fattening, as well as the three stages overall as the main indices to evaluate the productive performance of the animals (45). Tables 13, 14 present the results of these indices for broiler chickens supplemented with *P. aduncum*, *M. citrifolia*, and *A. altilis* EE at 0.005 and 0.01% of the diet.

**Table 13:** The variance analysis of the performance indices for broiler chickens supplemented with *M. citrifolia*, *P. aduncum*, and *A. altilis* leaves EE.

Performance indices	Broiler stage	*p*-value	VC (%)	Adjusted. *R*^2^ (%)
Weight Gain (gr/chicken)	Starting	0.0044	7.06	41.1
	Growth	0.0365	5.51	26.85
	Fattening	0.0078	7.94	37.62
	Total	0.0022	5.05	44.88
Feed Conversion	Starting*	0.0001	9.85	60.64
	Growth+	0.1516	8.00	13.99
	Fattening	0.0333	10.04	27.57
	Total	0.0302	7.85	28.31
Feed Consumption (gr/day/chicken)	Starting	0.0001	4.26	78.63
	Growth	0.0921	7.76	18.94
	Fattening*	0.4264	0.01	1.03
	Total*	0.4264	0.01	1.03
Final Weight (gr/chicken)	Starting	0.0005	5.68	51.9
	Growth	0.0056	4.89	39.62
	Fattening	0.0017	4.95	46.17
Carcass	Weight	0.0073	7.11	38.06
	Yield	0.7545	1.66	0.00

** and * Data were transformed with the base 10 logarithm and Box-Cox λ = −2, respectively.

**Table 14:** The performance indices of broiler chickens supplemented with 0.005 and 0.01% EE of *M. citrifolia*, *P. aduncum*, and *A. altilis* in the diet.

Productive indices	Rearing stages	Treatments							
		Control		*P. aduncum*		*M. citrifolia*		*A. altilis*	
		−	+	0.005	0.01	0.005	0.01	0.005	0.01
Weight gain (WG) (gr/chicken)	Starting	129.66b	133.73b	129.27b	135.80b	153.27a	149.64	130.57b	128.30b
	Growth	699.79b	726.71	697.28b	729.94	797.83a	752.20	713.62	722.23
	Fattening	1117.14ab	1041.32b	1115.97ab	1061.62b	1112.18ab	1245.24a	962.27b	1041.03b
	Total	1946.59bc	1901.76bc	1942.52	1927.35bc	2063.28	2147.08a	1806.46c	1891.55bc
Feed conversion rate (FCR)	Starting*	1.46b	1.46 b	1.51b	1.46 b	1.37 b	1.41b	1.48 b	1.93a
	Growth^a^	1.61	1.47	1.54	1.49	1.44	1.54	1.52	1.48
	Fattening*	2.05a	2.20a	1.98a	2.10a	2.03a	1.78b	2.22a	2.17a
	Total*	1.87a	1.90a	1.79	1.83	1.76	1.67b	1.89a	1.88a
Feed intake (FI) (gr/day/chicken)	Starting	27.15c	27.97	27.80	28.30	29.98b	30.13b	27.68	35.65a
	Growth^+^	80.13	76.14c	76.42c	76.75bc	82.40ab	82.93a	78.54c	75.60c
	Fattening^a^	190.93	190.93	184.39	188.24	188.64	184.48	181.01	190.69
	Total^a^	109.38	107.56	105.5	107.38	109.78	108.6	104.52	108.99
Final Weight (FW) (gr/chicken)	Starting	169.11b	171.91b	167.61b	175.43b	196.98a	193.61a	168.73b	167.16b
	Growth	868.91b	898.62b	864.89b	905.38b	994.81a	945.82ab	882.35b	889.39b
	Fattening	1986.05bc	1939.94bc	1980.86bc	1966.99bc	2106.99ab	2191.06a	1844.62c	1930.42bc
Carcass yield (CY)	Weight	1631.67bc	1626.25bc	1689.25abc	1641.50bc	1874.00a	1861a	1526.50c	1797.75ab
	Yield	78.73	79.86	79.11	80.02	80.22	79.21	79.36	79.72

abcd: different letters denote significant differences between treatments, SNK test (*p* < 0.05). ^+^, Kruskal–Wallis ANOVA was applied, and medians are presented; *, Box-Cox with lambda −2 transformation; ^a^, no parametric tests were applied without ANOVA F modification.

The final weight for the fattening stage, the weight gain for this stage, and the three stages overall, respectively, were greater among chickens supplemented with 0.01% *M. citrifolia* EE than those in the positive control, negative control, and those supplemented with *P. aduncum* and *A. altilis* EE at 0.005 and 0.01% of the diet (*p* < 0.05).

In accordance with these indices, the FCR was lower in the fattening phase and for the three stages overall in chickens supplemented with 0.01% *M. citrifolia* EE than those in the positive and negative control, and those supplemented with *P. aduncum* and *A. altilis* EE at 0.005 and 0.01% of their diet (*p* < 0.05) (Tables 13, 14).

## Discussion

The objective of this study was to determine the effect of the ethanolic extracts (EE) of *P. aduncum*, *M. citrifolia*, and *A. altilis* on the intestinal health of broiler chickens. Previous studies have shown that *Piper aduncum*, *Morinda citrifolia*, and *Artocarpus altilis* possess a variety of phytochemicals, such as phenolic, triterpene, flavonoid, and phenylpropanoid compounds, which in general possess antibacterial (26–28), antioxidant, and anti-inflammatory properties (29–32). These properties of the three studied plants mainly those of *M. citrifolia*, might have increased glucose and triglycerides in blood of broiler chickens, decreased the staphylococcus abundance in the broiler’s microbiota, and increased crypt depth, villus width, and villi length to crypt depth ratio in the intestinal mucosa structure of broiler chickens in the present study.

### Hematology and metabolites profiles

Few studies have been published on the effects of extracts or essential oils from *P. aduncum*, *M. citrifolia*, and *A. altilis* on the hematological profiles of birds. The results obtained in the present study were like those reported in previous studies on birds (49), rats, and mice (Schuktz et al., 2017); (50–53), where similar hematological profiles were obtained on increase in the levels of *P. glabratum*, *P. aduncum*, *M. citrifolia*, and *A. altilis* extracts. However, few previous studies evaluating leaf powders of plants, such as *Moringa oleifera* and *Azadirachta indica*, demonstrated an increase in the hematological profiles of the broiler chickens (46, 54). The difference between the results obtained in the present study, and these previous results can be explained by the high protein and amino acid contents and the diverse nutritional components of *Moringa oleifera*, which may have contributed to the modulation of hematological responses in the birds (46, 55).

Few previous studies have been published on the effects of extracts or chemical fractions of *P. aduncum*, *M. citrifolia*, and *A. altilis* on blood metabolite profile. To our knowledge, triglyceride, AST, ALT, PT, albumin, and globulin in chickens supplemented with *A. altilis* EE have not been previously reported. Glucose is the primary form of energy obtained from different sources of carbohydrates in animals, mainly in birds (56). However, blood glucose levels in birds are 1.5 to 2 times greater than those in mammals (57, 58).

In contrast to mammals, the levels of insulin circulating in adult birds are approximately one-tenth of the levels found in rats (59). In the present study, the increase in glucose levels with age observed on supplementation with 0.01% *M. citrifolia* and *A. altilis* EE in 21 and 28 days of age, respectively, when compared with the negative and positive controls (Table 7), could be associated with antimicrobial effects; increased villi length and width and increased Lieberkühn crypt depth in the broiler chickens obtained in the present study.

In previous studies performed in rats treated with a fraction of *A. altilis* ethyl acetate and in others fed fruit-based diets of *A. altilis*, the blood glucose levels were reduced (50, 60), whereas these levels are similar in mice and rats treated with extracts from *M. citrifolia* fruit (51, 52).

Triglycerides are lipids synthesized by the hepatic tissue and are present at the highest quantities in vertebrates, including birds, and their main role is to serve as an energy reserve (58). The increase in triglyceride levels on supplementation with 0.01% *M. citrifolia* and *P. aduncum* EE in 28 days-old chickens (Table 8) might be associated with an increase in blood glucose level and an improved performance of the hepatic tissue as a result of the antioxidant and hepatoprotective effects of EE of these plants, particularly *M. citrifolia* (32, 61), which would allow for improved synthesis physiology in this organ.

Nonetheless, in previous studies in rats, triglyceride levels were unaffected after treatment with *P. aduncum* essential oil, like the results obtained for glucose, AST, and ALT levels (53, 62). A similar study has shown that the use of *M. citrifolia* fruit extract at different doses does not alter the triglyceride, AST, or ALT levels in chickens (63).

### Antimicrobial activity

Gut microbiota in poultry comes from exogenous microorganisms immediately after hatching, and thereafter, it shelters a microbial community, primarily anaerobic bacteria, which reaches a relatively stable dynamic state as the host grows (64). Most of the microbes in the intestinal microbiota of poultry in cultivation-based studies have been identified as Gram-positive rods and cocci (86%), followed by Gram-negative rods (14%) (65, 66). More recent studies using 16S rRNA methodology reveal that in the chicken intestinal microbiota predominate the phyla: Firmicutes (50%), Cyanobacteria (26%), and Proteobacteria (17%) (66, 67); in the chicken, ileal microbiota predominate Firmicutes (64.15%), Bacteroidetes (22.15%), and Proteobacteria (4.26%) (68); moreover, the predominance of one phylum of bacteria between other factors is associated with gender and breed of chickens (69).

As the gut microbiota is the microbial community, including commensal, symbiotic, and potential pathogenic microorganisms, which usually colonize the gut of animal organisms, the different kinds of additives including plant essential oils and extracts that regulate the intestinal microbiota directly regulate all these microorganisms (70). In addition, the regulated commensal and symbiotic intestinal microbiota compete with the colonizing potential pathogenic bacteria and can reduce the adhesion and colonization of pathogens in the intestine of chickens (64, 71), and by these mechanisms, the EEs might regulate the chicken microbiota and improve the intestinal health.

The antimicrobial activity of an extract or essential oil is influenced by its chemical structure, the presence of different functional groups, concentration, and possible synergistic or antagonistic effects between the components of the extract or oil (24, 72). Antimicrobial activity of the extracts or essential oils from plants is primarily attributed to phenols, and the phenol concentration in a plant determines its antimicrobial potential (18, 73, 74). In the previous phase of this study, polyphenols between 150.8 and 1250.4 mg/100 g and flavonoids between 1.8 and 30.7 mg/100g were determined for the *P. aduncum*, *M. citrifolia*, and *A. altilis* leaf-dried EE.

The decrease in the Gram-positive population (log_10_CFU), such as *Staphylococcus. aureus*, in the intestinal content of the broiler chickens on dietary supplementation of 0.01% *M. citrifolia* EE concurred with the minimum inhibitory concentration (MIC) in our previous research, wherein 3.12 mg/mL of *M. citrifolia* EE inhibited the *in vitro* growth of *Staphylococcus aureus ATCC 25923*.

This effect of *M. citrifolia* EE in the intestines of chickens could have strengthened the mechanisms that the animals possess to limit microbial colonization in the intestinal crypts and glands (75), thus promoting an increase in the depth of the crypts and villi length to Lieberkühn crypt depth ratio obtained for the chickens in the present study.

These results highlight the antimicrobial activity of *M. citrifolia* EE against the intestinal microbiota of chickens, as previous studies have revealed that the microbiota is primarily composed of Gram-positive organisms (66). These results were supported by those obtained in previous studies, where it has been shown that phytochemical compounds in general have greater antimicrobial activity against Gram-positive bacteria since their antimicrobial mechanisms are linked to the hydrophobicity of the molecules, which enter into the single membrane covering, thus disrupting permeability and homeostasis, resulting in a consequent loss of the cellular components and eventual cell death (72, 76, 77).

In contrast, Gram-negative bacteria are more tolerant than Gram-positive bacteria to the action of phytochemical compounds because they possess an additional external membrane, which is almost impermeable to the hydrophobic molecules of phytocompounds (78, 79). This could explain the similar results obtained for *E. coli* and *Lactobacillus* sp. populations in the present study and supports the MIC results of our previous research using the EE from these three plants wherein the growth of *E. coli* ATCC 25922 and *Bacillus subtilis* ATCC 6633 strains were not inhibited.

In contrast, the reduction of *E.coli* population in the intestinal content of chickens has also been observed previously using essential oils from other plants or phytogenic additives (42, 43, 80–82). Moreover, previous research related to antimicrobial activity using extracts or essential oils from the three plants used in the present study is limited.

### Intestinal morphometry

The cells that cover the surface of the depths of the Lieberkühn crypts are pluripotent mother cells that differentiate into goblet cells, Paneth cells, enteroendocrine cells, and enterocytes, which migrate and mature to repair and replace those desquamated from the villi (83–85).

The development of these mechanisms of formation and function in the mucosa of the duodenum, jejunum, and ileum is one of the cornerstones of intestinal health, which can improve as the crypts increase in depth (86, 87). This increase was observed with age in the present study on supplementation with 0.01% *M. citrifolia* EE in 21 days of age chickens, where the intestinal crypts had the greatest depth, when compared with the results obtained for chickens from the positive and negative control groups (*p* < 0.05).

Similarly, the depth of the crypts increased with age on supplementation with 0.01% *P. aduncum* and the *M. citrifolia* in 21 and 28 days of age, respectively, compared with those of the positive control (*p* < 0.05). Previous studies using extracts from other plants have proven that the length of the intestinal villi increases because of plant extracts or essential oils (15, 16, 88–90), facilitating the mechanisms of nutrient absorption. This is similar to the active mechanisms of antibiotics as growth promoters, which also promote an increase in the length of the intestinal villi (5).

Notwithstanding, the increase with age in the depth of the crypts, the width of the villi, and the villi length to crypt depth ratio obtained in the present study on supplementation with 0.01% *M. citrifolia* and *P. aduncum* EE are consistent with previous research (86, 87, 91, 92). Increase in these mucosal structures increases nutrient absorption and enzyme production due to a more dynamic replacement mechanism for the enterocytes in the villi. This increase also promotes mechanisms that increases the population of goblet cells, which secrete mucus; Paneth cells present in birds (93), which secrete antimicrobial products such as lysozymes; and enteroendocrine cells which secrete local hormones (75, 94) in a balanced manner.

The integration of these mechanisms would result in a more integral strengthening of the mucosa epithelium functioning in the small intestine, with only the absorption produced by the increase in the length of the villi, as they are for secretion and barrier, which depend critically on the rapid renovation of epithelial cells, maintaining a balance between proliferation and cellular differentiation to support these functions of the small intestine (95, 96). Furthermore, the development of these mechanisms in the intestinal mucosa because of the leaves of *M. citrifolia* might be associated with the integration of antimicrobial (26, 27, 97) and antioxidant activities of this plant (29–31).

*M. citrifolia* fruit juice possesses 2.8 times the antioxidant activity of vitamin C, diminishing the blood levels of malondialdehyde and increasing those of superoxide dismutase, which are markers of the cells’ antioxidant defense system (29–32). The endogenous mechanisms of antioxidant activity, such as uric acid production in birds, and the species’ low production of reactive oxygen, superoxide, and hydrogen peroxide further supplement the antioxidant mechanisms (57, 98). Birds exhibit high levels of superoxide dismutase, superoxide isolators, as well as catalase and glutathione peroxidase (99).

These mechanisms would promote the multiplication and growth of crypts, which originate from pluripotent cells of different cellular groups on the intestinal mucosa. To our knowledge, this is the first study to demonstrate the effects of *P. aduncum*, *M. citrifolia*, and *A. altilis* EE on intestinal morphometry.

### Productive performance

The results of the present study were similar to those obtained in previous studies, wherein the productive indices of cattle, tilapia, and guinea pigs improved on using pulp and fruit extracts from *M. citrifolia* (100–102). Studies have been performed in chickens, where extracts from different plants, such as Indian frankincense, caraway (*Carum carvi* L.), cloves (*Syzygium aromaticum*), holy basil (*Ocimum sanctum*), and licorice have shown improved productive indices (15, 16, 88–90). Nonetheless, the results from the present study also contrast with those of previous studies, in which the productive performance indices of chickens did not vary on using of *M. citrifolia* leaf powder or different fruit extract concentrations (33, 63). On the other hand, this EE did not influence the daily feed consumption and carcass yield (*p* > 0.05), which are in line with previous studies where have been shown that the inclusion of plant extracts or essential oils as feed additives may positively or negatively influence the organoleptic characteristics of the diet such as aroma and taste (103, 104). Feed palatability is a critical factor influencing feed intake and, subsequently, animal performance. It can significantly affect the acceptance and consumption of specific feed components (90, 105). However, most studies have shown no significant change in feed intake caused by aromatic plants, plant extracts or EO additives, although growth was often enhanced and the feed conversion rate improved in healthy chickens (18). Those findings are in line with the findings obtained in the present study, where the EE did not influence the feed intake in the growing, fattening and on the three stages overall in the chickens supplemented with EEs compared to those from the negative and positive control groups. It might be explained because the studied plants have neither an irritating odor nor a pungent test and that poultry as birds might not be sensitive to flavor or test which made them more tolerant to exposure of adequate levels of these plants EEs.

These results could be used in the main time in practical applications, such as: (1) supplementing *M. citrifolia* EE in broilers chicken reared in small-scale farms for improving performance and at the same time to validate our findings; (2) to start developing studies on the ways of formulation of this EE to optimize its use in poultry; (3) valuating the culture of *M. citrifolia* by farmers in the tropical areas because its potential use in poultry wellbeing, health, and production; however, poultry breeders and farmers should be aware of some limitations such as: (1) few studies with these EEs have been still carried out; (2) the supplementation of the EE in feed has some difficulties for the EE compounds to homogenate, degradation in the feeders, and low speed of being absorbed by the gut because of the very small quantities to be used; (3) the supplementation in drinking water is easier for the EE compounds to homogenate, fast in being absorbed by the gut but very difficult to manage the supplementation by itself; (4) for optimizing supplementation of extracts by drinking water, it needs automatized watering system; and (5) economic aspects of this EE is still pendant for being determined.

Regarding economic aspects that imply a growth promoter in poultry production, it is generally accepted that using antibiotics as growth promoters in poultry diets, feed utilization efficiency can be improved on average by 2–5% (Ly-Zi, et al., 2020). Very scarce trials in this aspect have been published with plant extracts and essential oils. A trial study with oregano essential oil (OEO) supplementation in broilers allowed a reduction in energy levels by 1–2%. This will lead to reduced feed costs and increased economic benefit in poultry farms (106). In addition, studies to evaluate the costs of different methods to obtain feed additives from plants did find that solvent extraction and supercritical fluid extraction are superior to other extraction methods in terms of low cost (107, 108). In the present study, no economic aspects of the EEs were considered; however, as it is one of the first studies using these bioactive plants on the modulation of intestinal health in broiler chickens, it is worthy to consider future research directions related to carry out more studies with these plants to search for more findings related to the bioactivity of its phytochemical compounds on animal wellbeing, health, and production and its economic aspects to validate these EEs as growth promoters in poultry.

## Conclusion

Dietary supplementation with EE 0.01% *M. citrifolia* decreased the abundance of *Staphylococcus aureus* in the intestinal microbiota and increased the depth of the Lieberkühn crypts and the villi length to Lieberkühn crypt depth ratio in the intestinal mucosa of 21 days of age broiler chickens, indicating improved intestinal health. In addition, 0.01% *M. citrifolia* EE supplementation increased blood glucose and triglyceride levels at 21 and 28 days of age, respectively. These interactions increased the final weight, weight gain during the fattening stage, and the total for the three rearing stages and further decreased FCR. Thus, the results of this study demonstrate a beneficial effect of the supplementation of *M. citrifolia* EE in improving gut health and some production indices of broilers chicken. This study also showed that the EE of *P. aduncum*, *A. altilis*, and mainly *M. citrifolia* did not have a detrimental effect on any of the parameters evaluated, so it is postulated as a potential alternative to replace AGP in poultry. These results could be used in the main time in practical applications such as: (1) supplementing *M. citrifolia* EE in broilers chicken reared in small-scale farms for improving performance and at the same time to validate our findings; (2) to start developing studies on the ways of formulation of this EE to optimize its use in poultry; and (3) valuating the culture of *M. citrifolia* by farmers in the tropical areas because its potential use in poultry wellbeing, health, and production. However, further studies will be necessary to determine the phytocomponents and mechanisms by which this extract exerts these effects in broiler chicken.

## Data availability statement

The original contributions presented in the study are included in the article/supplementary material, further inquiries can be directed to the corresponding author.

## Ethics statement

The animal study was approved by the Ethics and Animal Wellbeing Committee from the Veterinary Medicine Faculty, Universidad Nacional Mayor de San Marcos. The study was conducted in accordance with the local legislation and institutional requirements.

## Author contributions

DP-L: Funding acquisition, Project administration, Writing – original draft. MS-V: Investigation, Methodology, Writing – review & editing. RP-C: Investigation, Methodology, Writing – review & editing. SM-C: Investigation, Methodology, Writing – review & editing. XB-B: Conceptualization, Methodology, Writing – original draft. UA-P: Data curation, Software, Writing – review & editing. RR-H: Conceptualization, Supervision, Writing – review & editing.
